# Daptomycin antimicrobial activity tested against methicillin-resistant staphylococci and vancomycin-resistant enterococci isolated in European medical centers (2005)

**DOI:** 10.1186/1471-2334-7-29

**Published:** 2007-04-18

**Authors:** Helio S Sader, Amy A Watters, Thomas R Fritsche, Ronald N Jones

**Affiliations:** 1 JMI Laboratories, North Liberty, Iowa, USA; 2 Universidade Federal de Sao Paulo, Sao Paulo, Brazil; 3 Tufts University School of Medicine, Boston, Massachusetts, USA

## Abstract

**Background:**

Daptomycin is a cyclic lipopeptide with potent activity and broad spectrum against Gram-positive bacteria currently used for the treatment of complicated skin and skin structure infections and bacteremia, including right sided endocarditis. We evaluated the in vitro activity of this compound and selected comparator agents tested against clinical strains of staphylococci and enterococci collected in European medical centers in 2005.

**Methods:**

A total of 4,640 strains from 23 medical centers located in 10 European countries, Turkey and Israel (SENTRY Program platform) were tested for susceptibility by reference broth microdilution methods according to Clinical and Laboratory Standards Institute guidelines and interpretative criteria. Mueller-Hinton broth was supplemented to 50 mg/L Ca^++ ^for testing daptomycin. Results for oxacillin (methicillin)-resistant staphylococci and vancomycin-resistant enterococci were analyzed separately.

**Results:**

Oxacillin resistance rates among *Staphylococcus aureus *varied from 2.1% in Sweden to 42.5% in the United Kingdom (UK) and 54.7% in Ireland (29.1% overall), while vancomycin resistance rates varied from 0.0% in France, Sweden and Switzerland to 66.7% in the UK and 71.4% in Ireland among *Enterococcus faecium *(17.9% overall). All *S. aureus *strains were inhibited at daptomycin MIC of 1 mg/L (MIC_50/90_, 0.25/0.5 mg/L; 100.0% susceptible) and only one coagulase-negative staphylococci strain (0.1%) showed an elevated (>1 mg/L) daptomycin MIC value (4 mg/L). Among *E. faecalis *(MIC_50/90_, 0.5/1 mg/L; 100% susceptible) the highest daptomycin MIC value was 2 mg/L; while among *E. faecium *(MIC_50/90_, 2/4 mg/L; 100% susceptible) the highest MIC result was 4 mg/L.

**Conclusion:**

Daptomycin showed excellent in vitro activity against staphylococci and enterococci collected in European medical centers in 2005 and resistance to oxacillin, vancomycin or quinupristin/dalfopristin did not compromise its activity overall against these pathogens. Based on these results and those of previous publications, daptomycin appears to be an excellent therapeutic option for serious infections caused by oxacillin-resistant staphylococci and vancomycin-resistant enterococci in Europe.

## Background

Gram-positive bacteria, especially staphylococci and enterococci, are extremely important pathogens causing infections in the hospital environment. *Staphylococcus aureus*, coagulase-negative staphylococci (CoNS) and enterococci are among the five most frequently isolated organisms from nosocomial bloodstream infections (BSI) [1,2]. These three pathogens are responsible for approximately one-half of the cases of BSI in North American medical centers evaluated by the SENTRY Antimicrobial Surveillance Program [1].

Oxacillin (methicillin)-resistant *S. aureus *(MRSA) is currently recognized as a major problem in hospitals worldwide [3]. The 2004 National Nosocomial Infections Surveillance (NNIS) system report identified methicillin resistance in 59.5% of *S. aureus *infections in intensive care unit (ICU) patients [4]. This represented an 11% increase in resistance compared with rates for the period 1998 to 2002. The SCOPE project report showed a significant increase in the proportion of *S. aureus *isolates resistant to methicillin among patients in ICUs from 1995 to 2001 (22% vs. 57%; *P *< 0.001) [5]. In hospitalized pediatric patients with staphylococcal BSI, the proportion expressing methicillin resistance increased from 10% in 1995 to 29% in 2001 [6]. Furthermore, MRSA has recently emerged as an important cause of community-acquired infection in many parts of the United States (USA) [7].

A dramatic rise in frequency of enterococcal infections and the prevalence of vancomycin-resistant enterococci (VRE) occurred during the 1990s in the USA, first in ICUs, then essentially throughout hospitals [8-10]. The 2004 NNIS report indicated that nearly 30% of all enterococci isolated from patients infected in ICUs were resistant to vancomycin [4]. Although most European countries were able to control the hospital dissemination of VRE in the 1990s, the prevalence of this pathogen has recently increased dramatically in many European countries [8,11].

Daptomycin is a cyclic lipopeptide derived from *Streptomyces roseosporus *[12-14]. The mode of action is unique in that it binds to bacterial membranes, in the presence of physiological levels of calcium ions [15,16]. Daptomycin is primarily effective against Gram-positive bacteria, due to its inability to penetrate the outer membrane of Gram-negative organisms [17]. It is available as an intravenous drug and exhibits linear kinetics and is rapidly bactericidal [12,16-18]. Daptomycin is active against a wide range of multidrug-resistant (MDR) organisms for which there are very few therapeutic alternatives, such as MRSA and VRE. Due to daptomycin's requirement of calcium to be present for effective binding to the bacterial membrane, susceptibility testing requires appropriate supplementation of calcium to simulate levels found in human serum (45–55 mg/L) [14-16].

Daptomycin was approved by the USA Food and Drug Administration (FDA) in September 2003 for the treatment of complicated skin and skin structure infections (cSSTI) at a dosage of 4 mg/kg every 24 hours; and more recently for the treatment of *S. aureus *bacteremia with or without right sided infectious endocarditis at a dosage of 6 mg/kg every 24 hours [12,17,19]. Daptomycin has also been recently approved by the European Medicines Agency (EMEA) for the treatment of cSSTI [20]. In addition, the European Committee for Antimicrobial Susceptibility Testing (EUCAST) has assigned daptomycin breakpoints for staphylococci and streptococci [21], which are ≤ 1 mg/L for susceptible (similar to Clinical and Laboratory Standards Institute [CLSI] and US-FDA breakpoints) and ≥2 mg/L for resistance [22].

We have recently published a comprehensive analysis of the antimicrobial susceptibility of Gram-positive bacteria collected in European medical centers in previous years (2002–2004) [11]. In the present study, we summarize the antimicrobial activity results of daptomycin and several comparator agents tested against 4,640 clinical staphylococcal and enterococcal isolates collected in 2005.

## Methods

### Bacterial strains

A total of 4,640 contemporary clinical isolates, including 2,887 staphylococci (1,946 *S. aureus *and 941 CoNS) and 953 enterococci (646 *Enterococcus faecalis *and 307 *E. faecium*), were evaluated in the present study. All isolates were non-duplicate, consecutive, clinical strains collected from patients hospitalized in 23 medical centers located in 10 European countries, Turkey and Israel in 2005 (SENTRY Program platform) following common protocols. The isolates were collected according to site of infection. Identifications were performed by the submitting laboratories and confirmed in the monitoring laboratory (JMI Laboratories, North Liberty, Iowa, USA) using standard biochemical algorithms and/or the Vitek System (bioMerieux, Hazelwood, Missouri, USA).

### Antimicrobial agents and susceptibility testing

Daptomycin and selected comparator agents were tested by CLSI (formerly the NCCLS) criteria [22]. All strains were tested for antimicrobial susceptibility by the broth microdilution method. Dry-form, validated microdilution panels and broth reagents were manufactured by TREK Diagnostics (Cleveland, OH, USA). Mueller-Hinton Broth (MHB) adjusted to contain physiological levels of calcium (50 mg/L) was used when testing daptomycin [15]. Comparator agents included those representing the most common classes and examples of drugs used for the empiric or directed treatment of the indicated pathogen.

The isolates were categorized as susceptible and resistant according to CLSI guidelines [22]. A daptomycin susceptible breakpoint of ≤ 1 mg/L was used for staphylococci, while ≤ 4 mg/L was used for enterococci, as approved by the USA-FDA, CLSI and EUCAST [12,20-22]. The following quality control organisms were concurrently tested: *E. faecalis *ATCC 29212, and *S. aureus *ATCC 29213.

## Results

The isolates were collected mainly from bloodstream infection (55.1%), skin and skin structure infections (22.3%) and pneumonia (8.1%). France (940 strains; 20.3% of all isolates), Germany (848 strains; 18.3%) and Italy (519 strains; 11.2%) contributed with the highest number of strains; while Greece (78 strains; 1.7%), Israel (187 strains; 4.0%) and Switzerland (217 strains; 4.7%) supplied the lowest number of samples (Table 1). The frequencies of oxacillin resistance among staphylococci and vancomycin resistance among enterococci by country are listed in Table 2, and showed that oxacillin resistance rates varied from 2.1% in Sweden (187 strains) to 42.5% in the United Kingdom (UK; 153 strains) and 54.7% in Ireland (203 strains) among *S. aureus *(29.1% overall). Oxacillin resistance rates were most elevated among CoNS strains, varying from 53.3% in the UK (30 strains) to 83.3% in Greece (24 strains). Vancomycin-resistant *E. faecalis *strains were detected only in Italy (one strain; 1.6%) and UK (five strains; 17.9%), while among *E. faecium*, the vancomycin resistance rates varied from 0.0% in France, Sweden and Switzerland to 66.7% in the UK (18 strains) and 71.4% in Ireland (14 strains), with an all Europe rate of 17.9% (Table 2).

**Table 1:** Number of strains tested from European nations in the 2005 surveillance sample.

	No. tested by country:												
	
Organism	France	Germany	Greece	Ireland	Israel	Italy	Poland	Spain	Sweden	Switzerland	Turkey	UK	Total
*S. aureus*	594	471	41	203	113	240	213	241	187	102	188	153	2,746
Oxacillin-susceptible	407	390	26	92	61	148	155	180	183	86	130	88	1,946
Oxacillin-resistant	187	81	15	111	52	92	58	61	4	16	58	65	800
													
CoNS	224	187	24	3	30	182	16	42	31	90	82	30	941
Oxacillin-susceptible	65	61	4	1	6	32	3	16	14	31	21	14	268
Oxacillin-resistant	159	126	20	2	24	150	13	26	17	59	61	16	673
													
*E. faecalis*	104	117	7	38	34	61	40	39	96	21	61	28	646
Vancomycin-susceptible	104	117	7	38	34	60	40	39	96	21	61	23	640
Vancomycin-resistant	0	0	0	0	0	1	0	0	0	0	0	5	6
													
*E. faecium*	18	73	6	14	10	36	23	14	21	4	70	18	307
Vancomycin-susceptible	18	61	5	4	6	29	22	12	21	4	64	6	252
Vancomycin-resistant	0	12	1	10	4	7	1	2	0	0	6	12	55
													
Total	940	848	78	258	187	519	292	336	335	217	401	229	4,640

**Table 2:** Frequency of important resistance phenotypes by European nation.

Country	MRSA^a^	MR-CoNS^a^	Vancomycin-resistant *E. faecalis*	Vancomycin-resistant *E. faecium*
France	31.5	71.0	0.0	0.0
Germany	17.2	67.4	0.0	19.7
Greece	36.6	83.3	0.0	16.7
Ireland	54.7	66.7	0.0	71.4
Israel	46.0	80.0	0.0	40.0
Italy	38.3	82.4	1.6	19.4
Poland	27.2	81.3	0.0	4.3
Spain	25.3	61.9	0.0	14.3
Sweden	2.1	54.8	0.0	0.0
Switzerland	15.7	65.6	0.0	0.0
Turkey	30.9	74.4	0.0	8.6
UK	42.5	53.3	17.9	66.7
Overall	29.1	71.5	0.9	17.9

a. MRSA = oxacillin-resistant *S. aureus *and MR-CoNS = oxacillin-resistant CoNS.

Daptomycin was generally very potent against the Gram-positive organisms collected in European medical centers in 2005 (Table 3). All *S. aureus *strains were inhibited at a daptomycin MIC of ≤1 mg/L (100.0% susceptible) with a MIC_50 _of 0.25 mg/L and a MIC_90 _of only 0.5 mg/L. A slight trend toward higher daptomycin MIC values was observed for MRSA (52.8% at 0.25 mg/L and 43.8% at 0.5 mg/L) compared to oxacillin (methicillin)-susceptible *S. aureus *(MSSA; 70.7% at 0.25 mg/L and 24.9% at 0.5 mg/L). This very modest skewing was less apparent for CoNS where the frequency of strains inhibited at 0.25 mg/L were 48.1 and 47.3%, and at 0.5 mg/L were 33.6 and 43.2% for oxacillin (methicillin)-susceptible (MS-CoNS) and oxacillin (methicillin)-resistant (MR-CoNS) strains, respectively. Only one CoNS strain (0.1%) exhibited an elevated daptomycin MIC value for an oxacillin-susceptible strain isolated from a medical center located in Rome, Italy (reproducible MIC value of 4 mg/L). All other CoNS strains were inhibited at daptomycin MIC ≤1 mg/L (Table 3).

**Table 3:** Frequency of occurrence of daptomycin MIC values for key Gram-positive pathogens.

	No. (%) of isolates inhibited at daptomycin MIC (mg/L) of:						
	
Organism (no. tested)	≤0.06	0.12	0.25	0.5	1	2	4
*S. aureus *(2,746)	3 (0.1)	90 (3.3)	1.798 (65.5)	835 (30.4)	20 (0.7)	-	-
Oxacillin-susceptible (1,946)	2 (0.1)	75 (3.9)	1,376 (70.7)	485 (24.9)	8 (0.4)	-	-
Oxacillin-resistant (800)	1 (0.1)	15 (1.9)	422 (52.8)	350 (43.8)	12 (1.5)	-	-
Coagulase-negative staphylococci (941)	13 (1.4)	72 (7.7)	447 (47.5)	381 (40.5)	27 (2.9)	-	1 (0.1)^a^
Oxacillin-susceptible (268)	6 (2.2)	30 (11.2)	129 (48.1)	90 (33.6)	12 (4.5)	-	1 (0.4)^a^
Oxacillin-resistant (673)	7 (1.0)	42 (6.2)	318 (47.3)	291 (43.2)	15(2.2)	-	-
*E. faecalis *(646)	2 (0.3)	2 (0.3)	34 (5.3)	303 (46.9)	291 (45.0)	14 (2.2)	-
Vancomycin-susceptible (640)	2 (0.3)	2 (0.3)	34 (5.3)	299 (46.7)	289 (45.2)	14 (2.2)	-
Vancomycin-non-susceptible (6)	-	-	-	4 (66.7)	2 (33.3)	-	-
*E. faecium *(307)	-	1 (0.3)	3 (1.0)	19 (6.2)	72 (23.5)	151 (49.2)	61 (19.9)
Vancomycin-susceptible (252)	-	1 (0.4)	3 (1.2)	18 (7.1)	60 (23.8)	120 (47.6)	50 (19.8)
Vancomycin-non-susceptible (55)	-	-	-	1 (1.8)	12 (21.8)	31 (56.4)	11 (20.0)

a. Single strain of MS-CoNS.

Daptomycin was also highly active against enterococci (Table 3). Among 646 tested *E. faecalis *(MIC_50_, 0.5 mg/L; MIC_90_, 1 mg/L; 100% susceptible) the highest daptomycin MIC value was only 2 mg/L (2.2% of strains tested); while among *E. faecium *(MIC_50_, 2 mg/L; MIC_90_, 4 mg/L; 100% susceptible) the highest MIC value was 4 mg/L.

MSSA strains showed high rates of susceptibility (>95%) to most comparison antimicrobial agents tested except erythromycin (85.9% susceptibility), ciprofloxacin (93.0%) and levofloxacin (93.9%). In contrast, resistance rates to many agents were high among MRSA strains (Table 4). The most active compounds tested against this pathogen (100.0% susceptible) were daptomycin (MIC_90_, 0.5 mg/L), linezolid (MIC_90_, 2 mg/L), teicoplanin (MIC_90_, ≤2 mg/L) and vancomycin (MIC_90_, 1 mg/L; Table 4). CoNS showed higher rates of resistance compared to *S. aureus*. The fluoroquinolones, ciprofloxacin and levofloxacin, were active against only 86.9 and 87.3% of MS-CoNS, respectively. In contrast, daptomycin, linezolid and vancomycin were the only compounds active against 100.0% of MR-CoNS at the susceptible breakpoint (Table 4). It is important to note the emergence of quinupristin/dalfopristin resistance among both MRSA (98.6% susceptible) and MR-CoNS (99.3% susceptible).

**Table 4:** Antimicrobial activity of daptomycin and selected comparators tested against European *S. aureus *and enterococcal isolates (2005).

	MIC (μg/ml):			% category	
		
Organism (no. tested)	50%	90%	Range	Susceptible	Resistant
*S. aureus*					
Oxacillin-susceptible (1,946)					
Daptomycin	0.25	0.5	≤0.06–1	100.0	-^a^
Ciprofloxacin	0.25	0.5	≤0.03–>4	93.0	6.2
Levofloxacin	≤0.5	≤0.5	≤0.5–>4	93.9	5.9
Erythromycin	0.25	>2	≤0.06–>2	85.9	13.6
Clindamycin	≤0.25	≤0.25	≤0.5–>2	97.1	2.7
Trimethoprim/sulfamethoxazole	≤0.5	≤0.5	≤0.5–>2	99.7	0.3
Quinupristin/dalfopristin	≤0.25	0.5	≤0.25–>2	99.8	0.1
Chloramphenicol	8	8	4–>16	98.4	1.4
Rifampin	≤0.25	≤0.25	≤0.25–>2	99.4	0.5
Teicoplanin	≤2	≤2	≤2–8	100.0	0.0
Vancomycin	1	1	≤0.12–2	100.0	0.0
Linezolid	1	2	0.25–2	100.0	-
					
Oxacillin-resistant (800)					
Daptomycin	0.25	0.5	≤0.06–1	100.0	-
Ciprofloxacin	>4	>4	0.06–>4	6.9	93.0
Levofloxacin	>4	>4	≤0.5–>4	6.8	91.1
Erythromycin	>2	>2	≤0.25–>8	24.0	74.9
Clindamycin	0.5	>2	≤0.25–>2	49.4	50.2
Trimethoprim/sulfamethoxazole	≤0.5	≤0.5	≤0.5–>2	94.1	5.9
Quinupristin/dalfopristin	0.5	1	≤0.25–>2	98.6	1.3
Chloramphenicol	8	16	≤2–>16	89.7	5.6
Rifampin	≤0.25	>2	≤0.25–>2	86.2	12.8
Teicoplanin	≤2	≤2	≤2–8	100.0	0.0
Vancomycin	1	1	0.25–2	100.0	0.0
Linezolid	1	2	0.12–2	100.0	-
					
Coagulase-negative staphylococci					
Oxacillin-susceptible (268)					
Daptomycin	0.25	0.5	≤0.06–4	99.6	-
Ciprofloxacin	0.12	4	≤0.03–>4	86.9	11.9
Levofloxacin	≤0.5	4	≤0.5–>4	87.3	10.4
Erythromycin	≤0.25	>8	≤0.25–>8	65.7	44.0
Clindamycin	≤0.25	≤0.25	≤0.25–>2	94.8	4.5
Trimethoprim/sulfamethoxazole	≤0.5	2	≤0.5–>2	91.8	8.2
Quinupristin/dalfopristin	≤0.25	≤0.25	≤0.25–1	100.0	0.0
Chloramphenicol	4	8	≤2–>16	97.4	2.6
Rifampin	≤0.25	≤0.25	≤0.25–>2	96.1	2.6
Teicoplanin	≤2	4	≤2–16	99.6	0.0
Vancomycin	1	2	≤0.12–4	100.0	0.0
Linezolid	1	1	0.12–2	100.0	-
					
Oxacillin-resistant (673)					
Daptomycin	0.25	0.5	≤0.06–1	100.0	-
Ciprofloxacin	>4	>4	≤0.03–>4	27.8	67.5
Levofloxacin	4	>4	≤0.5–>4	28.1	62.9
Erythromycin	>2	>2	≤0.06–>2	27.2	72.7
Clindamycin	<0.25	>2	≤0.25–>2	67.0	32.5
Trimethoprim/sulfamethoxazole	2	>2	≤0.5–>2	52.6	47.4
Quinupristin/dalfopristin	≤0.25	0.5	≤0.25–>2	99.3	0.4
Chloramphenicol	4	>16	≤2–>16	86.6	13.0
Rifampin	≤0.25	>2	≤0.25–>2	85.3	13.4
Teicoplanin	≤2	8	≤2–>16	97.3	0.6
Vancomycin	1	2	≤0.12–4	100.0	0.0
Linezolid	1	1	0.25–4	100.0	-
					
*E. faecalis*					
Vancomycin-susceptible (640)					
Daptomycin	0.5	1	≤0.06–2	100.0	-
Ampicillin	≤1	2	≤1–16	99.4	0.6
Ciprofloxacin	1	>4	0.12–>4	64.2	34.1
Levofloxacin	1	>4	≤0.5–>4	66.1	33.0
Gentamicin (HL)^b^	≤500	>1000	≤500–>1000	65.2	34.8
Streptomycin (HL)^b^	≤1000	>2000	≤1000–>2000	62.0	38.0
Chloramphenicol	8	>16	≤2–>16	68.9	30.4
Quinupristin/dalfopristin	>2	>2	≤0.25–>2	0.9	93.4
Teicoplanin	≤2	≤2	≤2	100.0	0.0
Linezolid	1	2	0.5–2	100.0	0.0
					
Vancomycin-resistant (6)					
Daptomycin	0.5	-	0.25–1	100.0	-
Ampicillin	≤1	-	2	100.0	0.0
Ciprofloxacin	>4	-	>4	0.0	100.0
Levofloxacin	>4	-	>4	0.0	100.0
Gentamicin (HL)^b^	>1000	-	<500–>1000	16.7	83.3
Streptomycin (HL)^b^	>2000	-	≤1000–>2000	16.7	83.3
Chloramphenicol	8	-	-	100.0	0.0
Quinupristin/dalfopristin	>2	-	>2	0.0	100.0
Teicoplanin	>16	-	>16	0.0	100.0
Linezolid	1	-	1–2	100.0	0.0
					
*E. faecium*					
Vancomycin-susceptible (252)					
Daptomycin	2	4	0.12–4	100.0	-
Ampicillin	>16	>16	≤1–>16	13.1	86.9
Ciprofloxacin	>4	>4	0.12–>4	7.5	78.2
Levofloxacin	>4	>4	≤0.5–>4	23.0	70.2
Gentamicin	≤500	>1000	≤500–>1000	63.1	36.9
Streptomycin	>2000	>2000	≤1000–>2000	40.1	59.9
Chloramphenicol	8	16	4–>16	83.1	7.3
Quinupristin/dalfopristin	0.5	>2	≤0.25–>2	70.2	17.9
Teicoplanin	≤2	≤2	≤2	100.0	0.0
Linezolid	1	2	0.25–2	100.0	-
					
Vancomycin-resistant (55)					
Daptomycin	2	4	0.5–4	100.0	-
Ampicillin	>16	>16	16–>16	0.0	100.0
Ciprofloxacin	>4	>4	1–>4	3.6	92.7
Levofloxacin	>4	>4	1–>4	7.3	92.7
Gentamicin (HL)^b^	>1000	>1000	≤500–>1000	47.3	52.7
Streptomycin (HL)^b^	>2000	>2000	≤1000–>2000	36.4	63.6
Chloramphenicol	8	16	≤2–>16	87.1	6.5
Quinupristin/dalfopristin	1	>2	≤0.25–>2	72.7	18.2
Teicoplanin	16	>16	≤2–>16	41.8	41.8
Linezolid	1	2	0.5–2	100.0	0.0

a. - = No breakpoint has been established by CLSI [22]or US-FDA [12].

b. HL = High level resistance

Daptomycin (MIC_90_, 0.5 mg/L; 100.0% susceptible), ampicillin (MIC_90_, 2 mg/L; 99.4% susceptible) and linezolid (MIC_90_, 2 mg/L; 100.0% susceptible) were very active against *E. faecalis*, and only six vancomycin-resistant *E. faecalis *strains (0.9%) were detected in Europe in 2005 (Table 4). These strains were from Italy (one strains) and the UK (five strains), and all six strains showed low daptomycin MIC values (0.25 – 1 mg/L). On the other hand, *E. faecium *exhibited high rates of resistance to most antimicrobials tested. Resistance to vancomycin was observed in 17.9% of *E. faecium *strains (Table 2) and only daptomycin (MIC_50_, 2 mg/L and MIC_90_, 4 mg/L) and linezolid (MIC_50_, 1 mg/L and MIC_90_, 2 mg/L) were active against all vancomycin-resistant *E. faecium *strains tested. Furthermore, only 72.7% of vancomycin-resistant and 70.2% of vancomycin-susceptible *E. faecium *strains were susceptible to quinupristin/dalfopristin (Table 4).

## Discussion

The treatment of serious MRSA infections presents a great challenge to clinicians, particularly bacteremias and infective endocarditis, for which bactericidal therapy is essential to maximize successful clinical outcomes [23,24]. Vancomycin has been the preferred antimicrobial agent to treat such MRSA infections; however, the clinical efficacy of this glycopeptide has become more limited [24-27]. In addition to the increasing reports of isolates with reduced susceptibility (vancomycin-intermediate *S. aureus *[VISA]) or high-level vancomycin resistance (vancomycin-resistant *S. aureus *[VRSA]), other reports have shown limited bactericidal activity against a large proportion of strains with vancomycin MIC values within the CLSI susceptible range [22,27-30]. Linezolid and quinupristin-dalfopristin represent alternative treatment options for serious MRSA infections; however, these compounds also possess important limitations. Quinupristin-dalfopristin, a streptogramin combination, requires a central venous access to be administrated and has been linked to some adverse events such as arthralgia and myalgia [31,32]. Concerns with linezolid, an oxazolidinone, include possible hematologic toxicity of long-term treatment and the fact that it is a bacteriostatic agent against staphylococci and enterococci and is not indicated for the treatment endocarditis and serious infections in immuno-suppressed patients [31,33].

Although they are relatively nonvirulent organisms, the enterococci have become increasingly common nosocomial pathogens because they are resistant to many antimicrobials and can survive in the environment for prolonged periods of time. Enterococci are intrinsically resistant to multiple antimicrobial agents. Furthermore, other agents that are active in vitro, such as vancomycin, are not bactericidal at clinically achievable concentrations. As shown in the present study, *E. faecium *is usually resistant to ampicillin, and this type of resistance has been reported as a significant predictor of treatment failure [9,34]. Combination therapy of a cell-wall active agent plus an aminoglycoside has become the "standard of care" for patients with serious enterococcal infections, such as endocarditis or BSI, but the prevalence of high-level resistance to aminoglycosides and to ampicillin are increasing, leaving glycopeptides as the remaining class of active antimicrobials. Clearly, the emergence of VRE has further complicated therapeutic options [35].

Two antimicrobial agents have been approved specifically for the treatment of VRE infections: quinupristin/dalfopristin and linezolid [31,36]. However, quinupristin/dalfopristin MIC_90 _results (16 mg/L) for *E. faecalis *systemic infections exceeds the maximum achievable serum concentrations, making this compound inactive for *E. faecalis *[9,32], and resistance among *E. faecium *has been recently increasing, especially in Europe among vancomycin-resistant strains [11,35,37]. In this presented study, only 70% of *E. faecium *were susceptible to quinupristin/dalfopristin. On the other hand, linezolid has potent in vitro activity against both vancomycin-resistant *E. faecalis *and *E. faecium*, as well as good therapeutic efficacy for VRE bacteremia in mice [31,33,34]. However, as mentioned previously, linezolid has not been recommended for the treatment of endocarditis or serious infections in immuno-suppressed patients due to its predominantly bacteriostatic activity. In addition, the emergence of oxazolidinone resistance has been reported, especially in patients who receive prolonged courses of therapy [33,38].

## Conclusion

Daptomycin is the first member of a novel class of antimicrobial agents, the cyclic lipopeptides [13,14]. It has broad-spectrum and potent bactericidal activity against Gram-positive pathogens, including MRSA and VRE [39-42]. This compound has demonstrated activity against both growing and stationary-phase bacteria [12-14], [16-18]. Here daptomycin was recognized as highly active against *S. aureus *and CoNS, including oxacillin-resistant strains. Only one strain showed a daptomycin MIC value >1 mg/L (a CoNS strain with MIC value of 4 mg/L). Vancomycin (MIC_50_, 1 mg/L) and linezolid (MIC_50_, 1 – 2 mg/L) were active against all staphylococcal strains tested. However, daptomycin (MIC_50_, 0.25 – 0.5 mg/L) was four-fold more potent than these key comparison bacteriostatic antimicrobials. Furthermore, all enterococci, including vancomycin-resistant strains were susceptible to daptomycin and resistance rates were relatively high to all other compounds tested, except linezolid (see Table 4).

In summary, daptomycin showed excellent in vitro activity in a surveillance study against staphylococci and enterococci collected in European medical centers in 2005. Resistance to oxacillin, vancomycin, quinupristin/dalfopristin did not compromise daptomycin potency against these pathogens. Based on these results and those of previous publications [11,43,44], daptomycin appears to be an excellent therapeutic option for serious infections caused by MRSA, MR-CoNS and VRE (especially *E. faecium*) in Europe.

## Abbreviations

BSI – bloodstream infection

CLSI – Clinical and Laboratory Standards Institute

CoNS – coagulase-negative staphylococci

cSSTI – complicated skin and soft tissue infection

EMEA – European Medicines Agency

EUCAST – European Committee for Antimicrobial Susceptibility Testing

FDA – Federal Drug Administration

ICU – intensive care unit

MDR – multidrug-resistant

MHB – Mueller-Hinton broth

MIC – minimum inhibitory concentration

MR-CoNS – methicillin-resistant coagulase-negative staphylococci

MRSA – methicillin-resistant *Staphylococcus aureus*

MS-CoNS – methicillin-resistant coagulase-negative staphylococci

MSSA – methicillin-susceptible *Staphylococcus aureus*

NCCLS – National Committee for Clinical Laboratory Standards

NNIS – National Nosocomial Infections Surveillance

UK – United Kingdom

USA – United States of America

VISA – vancomycin-intermediate *Staphylococcus aureus*

VRSA – vancomycin-resistant *Staphylococcus aureus*

VRE – vancomycin-resistant enterococci

## Competing interests

H. S. Sader, T.R. Fritsche and R. N. Jones have received research/education grants in the last three years from: AB BIODISK, Abbott, AlamX, Arpida, AstraZeneca, Avexa, Basilea, Bayer, Becton Dickinson, bioMerieux, Bristol-Myers Squibb, Cadence, Cerexa, Chiron, Cubist, Daiichi, Elan, Elanco, Enanta, GlaxoSmithKline, Intrabiotics, Johnson & Johnson, LG Chemicals, Merck, Micrologix, Novartis, Optimer, Ordway, Osmotics, Peninsula, Pfizer, Replidyne, Schering-Plough, Sequoia, Serenex, Shionogi, Theravance, TREK Diagnostics, and Wyeth.

## Authors' contributions

HSS: Data analysis and manuscript writing.

AAW: Technical support and manuscript writing.

TRF: Data analysis and manuscript revision.

RNJ: Study design, data analysis and manuscript revision.

The content has been read and approved by all co-authors.

## Pre-publication history

The pre-publication history for this paper can be accessed here:


